# Comparison of Conventional Root Tip Resection with Digitally Guided Resection—An In Vitro Study

**DOI:** 10.3390/dj13100464

**Published:** 2025-10-10

**Authors:** Paul Kübel, Aydin Gülses, Juliane Wagner, Cedric Hinrichs, Jörg Wiltfang, Johannes Spille

**Affiliations:** Department of Oral and Maxillofacial Surgery, University-Hospital Schleswig-Holstein, Campus Kiel, 24105 Kiel, Germany

**Keywords:** surgical navigation, apicoectomy, endodontics, surgery, oral

## Abstract

**Background/Objectives**: In oral and maxillofacial surgery, apicoectomy is a standard procedure for treating persistent periapical infections after insufficient conservative treatment. Traditional techniques rely on direct visualization, while navigated methods offer advantages in precision and safety. This in vitro study compared conventional apicoectomy with dynamically guided navigation. The aim was to assess the feasibility, accuracy, and safety of dynamic navigation and to determine whether it reduces complication risks, improves surgical predictability, and minimizes bone loss. **Methods**: Ten experienced surgeons performed both techniques on custom-designed models. Operation time was assessed, as well as cavity volume, resected root length, incision width and height, and preservation of adjacent structures. **Results**: The navigated approach demonstrated significantly improved accuracy in root-end resection, with a reduction in access cavity volume (*p* < 0.001). No significant differences were found in operation time (*p* = 0.499), resection length (*p* = 0.054), or incision dimensions (*p* > 0.05). The risk of damaging adjacent structures was not significantly different between the two methods. **Conclusions**: Dynamic navigation for apicoectomy can offer an alternative in cases requiring high precision to conventional techniques. However, the routine clinical implementation of dynamic navigation remains limited due to the extensive preoperative planning required. The necessity for additional planning increases complexity, time, and cost.

## 1. Introduction

Apicoectomy is a well-established procedure in oral and maxillofacial surgery for treating persistent periapical infections by surgically removing the tip of the root, particularly when conventional endodontic therapy or endodontic retreatment fails [1,2]. The number of resections performed has remained consistent over the last few years. In Germany, approximately 521,000 root canal resections were reported to state health insurance funds in 2022 [3]. Traditionally, this procedure is typically performed under direct visualization, ideally with optical magnification aids, which can increase precision and reduce the risk of damage to adjacent anatomical structures [4]. Potentially damaged structures include the inferior alveolar nerve, neighboring roots, and the maxillary sinus; damage to these can cause altered sensation, numbness, and sinus infections and necessitate further treatments of neighboring teeth [5,6].

Conventional two-dimensional radiology is the standard for diagnosing and planning the therapy of endodontic lesions but is limited, especially in complex cases, due to visualization constraints [7,8]. The introduction of cone-beam computed tomography (CBCT) in the early 1970s revolutionized preoperative planning by providing three-dimensional imaging of the surgical site [9]. In the context of endodontic treatment, this allowed for improved localization of the apex, better preoperative visualization, enhanced diagnosis, and more accurate assessment of surrounding anatomical structures, such as the maxillary sinus or inferior alveolar nerve, compared to conventional 2D imaging [7,8].

This development has opened a new approach to endodontic microsurgery with static and dynamic navigation systems that have been successfully used in various maxillofacial surgeries as well as in implant dentistry [10,11]. It should be noted that a distinction must be made between static and dynamic navigation. Static navigation is characterized by a pre-operatively planned template that specifies a surgical procedure. Dynamic navigation, on the other hand, uses real-time tracking to compare 3D data with the surgical site and visualize the surgical plan. Static navigation, by utilizing preoperatively fabricated surgical guides, offers enhanced precision in osteotomy and root resection but lacks intraoperative flexibility [12]. Thanks to dynamic navigation, surgeons can adapt their procedures without losing the benefits of the guidance system and without the template there is always a full vision on the surgical side. Furthermore, dynamic navigation systems provide real-time tracking of surgical instruments based on CBCT data with optical tracking, which can improve intraoperative accuracy and flexibility.

Another aspect of significant relevance is artificial intelligence. This is developing rapidly and can already be used to support imaging, training, navigation planning, and the creation of surgical templates. In recent years, there has been continuous development in its areas of application across a wide range of medical disciplines [13,14,15,16,17,18].

Recent studies have demonstrated that dynamic navigation systems (DNS) reduce access cavity volume, minimize bone removal, and enhance resection accuracy, thereby preserving more healthy tissue while ensuring complete removal of the infected apex [19,20,21]. Despite these advantages, dynamic navigation is not yet fully implemented in routine clinical practice, primarily due to its demanding preoperative planning, the need for specialized equipment, and the associated learning curve for surgeons [22].

This study aims to evaluate whether the use of dynamic navigation, compared to a conventionally performed apicoectomy, can lead to improvements in accuracy, operation time, bone loss, and incision guidance. The apicoectomy was performed in a split-mouth design, either conventionally freehand or with the support of a dynamic navigation system, and was carried out by trained oral, maxillofacial, and facial surgeons. The hypothesis is that dynamic navigation allows for a minimized surgical approach, less damage to surrounding critical structures, and a better surgical outcome in terms of resected root length.

## 2. Materials and Methods

### 2.1. Study Design

Ten experienced oral and maxillofacial surgeons from the Department of Oral and Maxillofacial Surgery, University Medical Center Schleswig-Holstein, Campus Kiel, participated in the study. Ever surgeon is experienced in the traditional apicoectomy and are familiar with the system used, even if it is not used routinely. The procedure and the parameters were explained. Standardized instruments were used like in the daily clinical practice. Each performed both conventional and navigated apicoectomies on standardized models. Every surgeon performed two apicoectomies on the mesial root of the first molar per jaw, one using conventional techniques and one using dynamic navigation. Each participant was informed about the test procedures. The order of procedures was randomized to minimize learning bias. In the conventional group, the surgical procedure followed standard microsurgical principles. A semilunar flap was raised to expose the apical region using a 15c scalpel blade, followed by an osteotomy using a high-speed handpiece W&H Implantmed Plus SI-1023 (W&H Dentalwerk Bürmoos GmbH, Bürmoos, Germany) and a surgical bur with the diameter of 2.3 mm (Hager & Meisinger GmbH, Neuss, Germany). The root apex had to be resected at a 90° angle with a Lindemann milling cutter (Hager & Meisinger GmbH, Neuss, Germany), and the simulated cavity with granulation tissue had to be removed with a dental excavator. In the navigated group, the procedure incorporated real-time tracking, where the patient-specific surgical plan was loaded into the DENACAM^®^ system (mininavident AG, Basel, Switzerland). An optical tracking system registered the instruments with a registration tool and displayed the instrument on the computer screen relative to the CBCT-derived three-dimensional coordinates. With the help of navigation, the surgeons were able to display the planned traction cavity on the gingiva using the navigation system and thus plan the incision. Surgeons performed the osteotomy and root-end resection following real-time guidance displayed on the monitor. The remaining procedural steps and the used instruments were identical to the conventional method.

**Figure 1:**
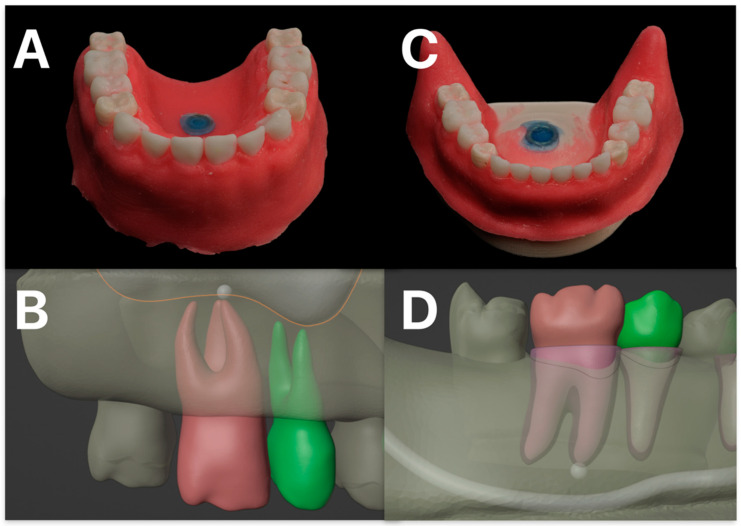
Modell design. Depicted in (**A**) is the 3D-printed maxillary model, and in (**C**) the corresponding mandibular model. The simulated gingival mask is clearly illustrated. In (**B**), the model planning for the maxillary molar and second premolar, including the respective roots and the maxillary sinus, is shown. The target structure is marked, representing a simulated periapical radiolucency. (**D**) illustrates the corresponding planning for the mandible, with the inferior alveolar nerve identified as an adjacent anatomical structure at risk.

The model depicted the roots of upper and lower first molar teeth and the roots of the second premolar embedded in a realistic bone model that replicated human maxillary and mandibular anatomy. Important neighboring structures such as the maxillary sinus, roots of neighboring teeth and the inferior alveolar nerve were also shown. The cancellous bone was imitated by a honeycomb-like structure. Important The model was 3D designed and printed by MedNerva GmbH (Limburg, Germany) (Figure 1). The selection criteria for the model ensured homogeneity in tooth positioning, root morphology, and surrounding bone density to eliminate potential confounding variables. The model is true to scale and based on real patient physiology in terms of anatomy. The model was designed so that it can be placed on a dental treatment chair.

**Figure 2:**
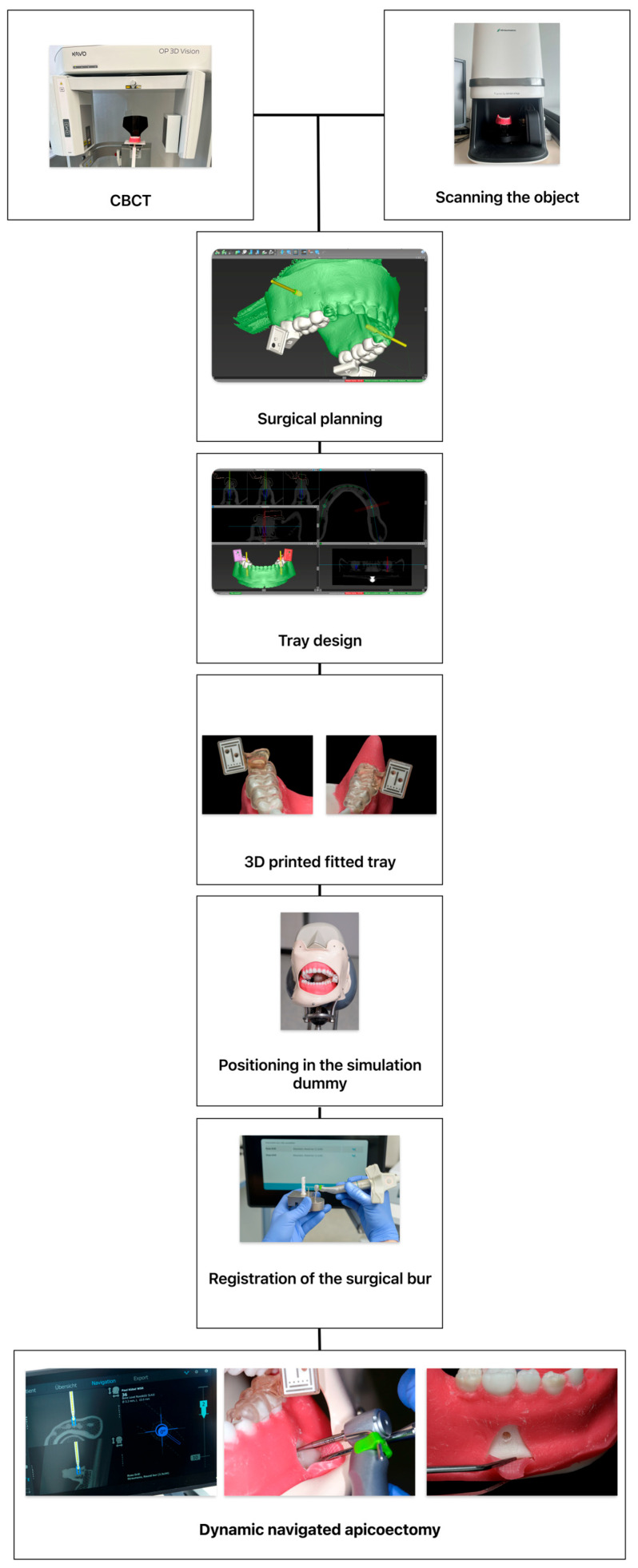
The figure illustrates the digital planning workflow. Firstly, acquisition of the cone-beam computed tomography (CBCT) scan and object scan of the model are made. Then surgical planning, digital placement of the reference marker, and design of the customized registration tray are followed. After 3D printing, the tray’s fit is verified on the model before being positioned within the simulation dummy. Prior to initiating navigated surgery, the surgical burr must be registered using a dedicated registration tool. On the navigation screen, the left side displays the real-time position of the burr overlaid on axial and coronal CBCT slices, while the right side of the display shows the target marker, the planned entry point, and the current drilling depth. In the center picture, the drilling procedure using a 2.3 mm surgical round burr is shown. On the right picture, the arc-shaped incision following apicoectomy is depicted.

**Figure 3:**
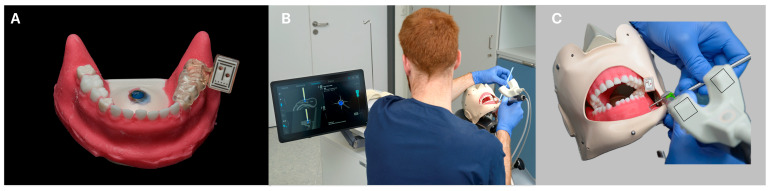
Illustrated workflow. In (**A**), the mandibular with the mounted individual printed tray with the marker is shown and (**B**) shows the operation with the help of the real-time navigation (**C**) is visualizing the two cameras of the DENACAM^®^ system (mininavident AG, Basel, Switzerland) by two squares.

Preoperative imaging was performed using the CBCT OP 3D Vision (KaVo Dental GmbH, Biberach, Germany) to obtain high-resolution, three-dimensional imaging of the surgical site. The scan was performed with a resolution of 0.2 mm voxel. The CBCT scans provided important anatomical details, including the position of the root apex, adjacent structures, and bone thickness, allowing precise planning of the apicoectomy procedure. Furthermore, the surface of the model was scanned with the object scanner Dental Wings^®^ 7SERIES (Dental Wings GmbH, Chemnitz, Germany). For the dynamic navigation group, the CBCT and model scan data were imported into the coDiagnostiX^®^ software (Dental Wings GmbH, Version 10, Chemnitz, Germany), where a virtual surgical plan was created simulating an implantation. This plan included the optimal osteotomy trajectory and targeted resection depth. The marker for the DENACAM^®^ system (mininavident AG, Basel, Switzerland) was virtually positioned, and a custom tray was fabricated using a resin printing process with a Formlabs 2 3D printer (Formlabs GmbH, Berlin, Germany) to serve as a marker holder (Figure 2 and Figure 3).

The Ethics Committee of the Kiel University has no objections to the ethical conduct of the study (D 564/20, on 18 August 2020) and a positive vote was received. All participating surgeons provided informed consent regarding data collection and analysis.

### 2.2. Variables and Data Collection Methods

Several parameters were assessed to evaluate the accuracy and efficiency of both techniques. Postoperative CBCT scans were acquired to compare the conventional versus guided resection in terms of resected root length and osteotomized volume, measured using the image analysis software 3D Slicer (https://www.slicer.org/, Version 5.6.1 [Computer Software] [23,24]). The total operation time was recorded from flap incision to completed resection. The surgical incision was measured in vertical and horizontal length, as were damaged neighboring structures like the inferior alveolar nerve, neighboring roots, or the maxillary sinus. Whether there was a non-bone-supported incision or if there was total resection of the planned root or damage to the root surface above the planned resection was considered.

### 2.3. Data Analysis

All data were analyzed using jamovi (The jamovi project (2024), Version 2.6 [Computer Software] [25]). For continuous variables, descriptive statistics were reported as the mean ± standard deviation and as frequencies for categorical variables. Group comparisons were performed using Student’s *t*-test for normally distributed parameters with homogeneity of variances. If the assumption of variance homogeneity was violated, the Mann–Whitney U test was used for non-normally distributed data, and the Chi-square test was used for categorical variables. A *p*-value of less than 0.05 was considered statistically significant. The evaluated parameters were operation time from incision to successful apicoectomy, vertical and horizontal incision length, whether the suture was bone-supported, resected root length, resected bone volume, and damaged root surface above the planned resection or damaged neighboring structures.

## 3. Results

**Figure 4:**
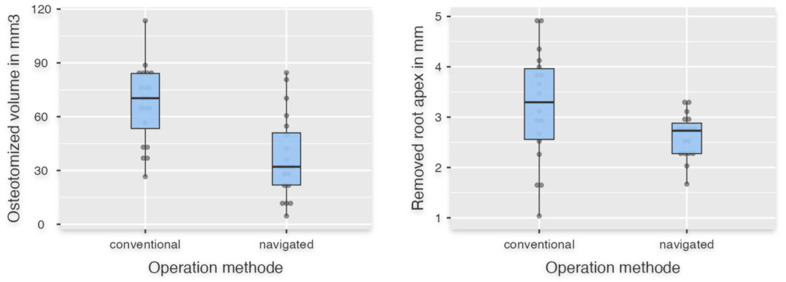
The left boxplot shows the data of osteotomized volume in the navigated and conventional groups. On the right the data of the resected root apex is shown.

**Figure 5:**
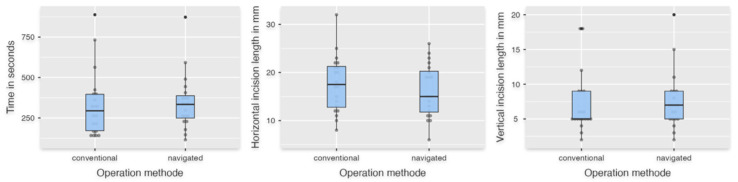
The left box plot shows the time in seconds. The middle plot shows the length of the cut in horizontal diameter, and the right plot shows the vertical height in millimeters.

Forty apicoectomies were performed by ten surgeons (navigated: *n* = 20; conventional: *n* = 20). For the descriptive statistics, mean values and standard deviations are shown in Table 1. Student’s *t*-test was used to analyze the parameters “Resected bone volume” (Figure 4) and “Horizontal incision length” (Figure 5). The resected bone volume with the navigated operation method was statistically significantly less than with the conventional method (*p* < 0.001) and showed a strong effect size (Cohen’s d = 1.342) (Table 2). For “Horizontal incision length,” there was no significant difference between FH and DNS (*p* = 0.442). The parameters “Time in seconds,” “Length of removed apex,” and “Vertical incision length” did not show a normal distribution and were therefore evaluated using the Mann–Whitney U test. The descriptive statistics showed fulfillment of the specification for the variable “Length of removed apex” of 2–3 mm of the root apex with 2.63 mm ± 0.44 mm compared to the conventional group with 3.21 mm ± 1.11 mm (Table 1 and Table 2; Figure 4). However, no significant difference was evaluated for “Time in seconds,” “Length of removed apex,” and “Vertical incision length” (*p*Time in seconds = 0.499; *p*Length of removed apex = 0.054; *p*Vertical incision length = 0.442) (Figure 5). In both groups, two tips were not completely removed due to a completely preserved root section in its original length and were therefore excluded from the parameter “Length of removed apex”.

The nominal scaled parameters “Completely removed root apex,” “Bone-supported incision,” and “Damaged root surface above” were evaluated with the Chi-square test with continuity correction and showed no statistical significance between the operation methods (*p*Completely removed root apex = 0.730; *p*Bone supported incision = 0.514; *p*Damaged root surface above = 0.405).

## 4. Discussion

Apicoectomy, a well-established surgical procedure following unsuccessful conservative endodontic treatment, has evolved into a minimally invasive technique utilizing magnification aids and microscopes [4]. Visualization and preoperative planning have been enabled by the subsequent development of guided surgery using 3D imaging [26]. As a result, it is now possible to choose between static and dynamic navigation. Navigation systems—developed for the oral cavity—have been used for dental implantation, with results comparable to, and in some cases better than, those achieved with conventional surgical methods [12,27,28]. For the implementation of a dynamically navigated digital workflow, the frame structures, corresponding devices, and 3D datasets must be created using CBCT and intraoral scanners. The primary objective was to facilitate navigation during dental implantation surgery. However, its applications extended to biopsies in cases of complex surgical anatomy or endodontic surgery [11,22,27,29,30].

For apicoectomy, both surgical approaches are described. For static navigation, Hawkins et al. described that individually planned and printed surgical guides reduced surgical time, resected less volume, and improved angulation, and Buniang et al. showed similar results to conventional root-end resection in a retrospective study [31,32]. Limitations in intraoperative adaptation were described, and the template had to be adequately supported dentally for high precision, but it has proven to work well in clinical cases [33,34,35].

The present study demonstrated the advantages of navigated apicoectomy using the DENACAM^®^ system (mininavident AG, Basel, Switzerland) in a patient-like setting with a difficult-to-view surgical site. The access volume was significantly reduced, and the required removal of 2–3 mm of the root apex could be demonstrated with greater precision than with the conventional method. This confirms our hypothesis that using the navigation system reduces the volume of the cavity. However, the secondary hypotheses regarding reduced root tip length, smaller incisions, and shorter operating times could not be confirmed. These results were comparable to the current state of research. Aldahmash et al. compared 24 apicoectomies utilizing DNS and 24 conventional apicoectomies in a cadaver study and showed significantly fewer deviations, angular deflection, reduced cavity volume, and shorter operating duration [19]. Similar results were found in the study by Dianat et al. for angular deflection, operating time, and linear deviation. Noteworthy was the subgroup with a thicker buccal cortical plate of more than 5 mm, which showed significantly lower accuracy and longer operating time in the conventional cohort, but not in the DNS cohort [20].

An advantage of our study was that the same approach was used for both resection procedures, with conventional surgical drills rather than pilot drills and then conical bone drills with a diameter of 3.5 mm or 4.2 mm, as in the studies described above. The use of a plastic jaw limits this setup. The material is softer during preparation, and the cortical structures cannot be adequately imitated. In addition, visualization is better in a dummy head without a tongue and saliva than in a patient or an cadaver [19].

Obligatory for navigated surgery is 3D imaging. The need for 3D imaging increases the patient’s exposure to radiation and must be critically scrutinized, even if studies showed that CBCT enabled better diagnosis of apical infections [7,8,36]. Antony et al. described in a systematic review that CBCT had the highest accuracy in detecting osteolysis of the bone due to periapical lesions [37]. In the study by Chugal et al., 442 scans evaluated 526 teeth, and CBCT led to a changed diagnosis of periapical lesions in 21% and a changed treatment plan in 69% [7].

Guided apicoectomy can assist with the limited field of view and more complicated handling situations in the molar region. To establish dynamic guidance systems, it is necessary to evaluate the practicality of the systems in a simulated clinical setting. Actual studies with human cadaver heads have shown good applicability as described above. In this study, we attempted to simulate a clinical environment. The designed and printed model allowed for a highly patient-like setup due to its design with patient-like jaw and dental anatomy, hard and soft tissues, fitting in a simulation dummy, and printed anatomical structures such as the maxillary sinus and adjacent roots.

Von Arx et al. showed that minimally invasive surgery is favorable for patient and tooth outcomes [4]. However, their focus was more on the development of tools like piezoelectric surgery and optical magnifiers. We need further prospective in vivo studies to investigate if a smaller volume defect and precise root resection influence the survival rate of treated teeth and the postoperative situation, such as pain and swelling. Important for the survival of teeth after apical surgery is the retrograde filling [38]. In this study, we focused primarily on the handling and outcome of dynamic navigated surgery. However, a smaller cavity can cause problems for curettage, hemostasis, and retrograde filling. Only the study from Aldahmash et al. showed the full operation with retrograde filling [19]. Due to the tapered 3.5 mm bur used, the cavity was always the same size and was suitable for retrograde treatment. Further studies should be conducted with navigation systems, and, in particular, with the DENACAM^®^ system (mininavident AG, Basel, Switzerland), to verify whether retrograde processing is also possible.

Furthermore, artificial intelligence represents a new development. It can assist with the diagnosis of periapical lesions, tooth cracks, and locating critical structures like the mandibular nerve [13,39,40]. The development of robot-assisted surgery, higher computer processing power, and artificial intelligence leads to the first fully automated dental surgeries [41,42]. Liu et al. showed in a study that a robotic system was significantly more accurate during apicoectomy than dynamic navigation in terms of platform and global apex deviation and angular deflection, and the compared operative time was longer with the robotic procedure than with the DNS procedure [43]. However, one problem with the robotic system is that it can only perform a resection. It is not possible to perform the entire procedure from incision to suturing. So, even though artificial intelligence and robotics in surgery are promising, we need more research and development. In summary, navigated surgery offers a more predictable outcome, a lower risk of complications, and greater safety, particularly for less experienced surgeons, but these benefits come at the cost of more planning compared to conventional surgery [11,21,22,26].

The present study concludes that the system investigated can be implemented for navigation in the workflow of a conventionally performed root tip resection. A reduction in the access volume was observed. No injuries to adjacent structures were found in either group. Likewise, no improvement in operating time or planned root tip length was observed. The incision in the navigated group was congruent with that in the conventional group. The analysis showed that the parameters “Complete removed root apex”, “Damaged root surface above” and “Bone-supported incision” also did not show any significant advantage.

The current study had also some major limitations. First, because this study was based on a small sample size, a larger study is needed to improve statistical power. Besides that, the model was produced from a plastic material, and due to the higher temperature at the tip of the bur, it was sometimes difficult to visually distinguish between the printed root and the bone material. Moreover, it is not possible to accurately compare the simulation conditions with real-life scenarios. Ultimately, this is purely an in vitro study. Real-life conditions, such as patient movement, salivation, pain and bleeding, cannot be sufficiently simulated. Further studies are required to determine whether the procedure from resection to retrograde filling can be implemented in a clinical setting. In the future, a large database or a prospective in vivo study for dynamic guided root resection should be established.

## 5. Conclusions

Dynamic navigation for apicoectomy can offer an alternative to conventional techniques, particularly in cases requiring high precision. The improved accuracy and reduced bone loss suggest potential clinical benefits. However, the routine clinical implementation of dynamic navigation remains limited due to the extensive preoperative planning required. The necessity for additional imaging, software-based planning, and intraoperative calibration increases complexity and cost, restricting its widespread use in daily clinical practice. Further studies are needed to assess the clinical workflow, optimize its efficiency, and evaluate clinical outcomes to enhance its feasibility for broader application.

## Figures and Tables

**Table 1:** Descreptive statistics for the operation method. Displayed are the number of attempts, means, and standard deviations.

	Operation Method	*n*	Mean Value	Standard Deviation
Horizontal incision length in mm	Conventional	20	17.65	5.91
	Navigated	20	16.25	5.48
Resected bone volume in mm [3]	Conventional	20	67.23	21.57
	Navigated	20	37.32	22.97
Length of removed apex in mm	Conventional	18	3.21	1.11
	Navigated	18	2.63	0.44
Time in seconds	Conventional	20	329.65	201.98
	Navigated	20	345.5	170.01
Vertical incision length in mm	Conventional	20	7.1	4.38
	Navigated	20	7.5	4.14

**Table 2:** Evaluation of the examined data using the appropriate statistical tests and effect strength.

	Test	*p*	Effect Strength	
Horizontal incision length	Student’s *t*	0.442	Cohens d	0.246
Resected bone volume	Student’s *t*	<0.001	Cohens d	1.342
Length of removed apex	Mann–Whitney U	0.054	Biserial rank correlation	0.380
Time in seconds	Mann–Whitney U	0.499	Biserial rank correlation	0.128
Vertical incision length	Mann–Whitney U	0.433	Biserial rank correlation	0.145

## Data Availability

The data presented in this study are available on request from the corresponding author due to privacy reasons.

## Abbreviations

The following abbreviations are used in this manuscript:

CBCT	Cone-beam computed tomography
DNS	Dynamic navigation systems
FH	Free-handed

## References

[1] Dioguardi M., Stellacci C., La Femina L., Spirito F., Sovereto D., Laneve E., Manfredonia M.F., D’Alessandro A., Ballini A., Cantore S. (2022). Comparison of Endodontic Failures between Nonsurgical Retreatment and Endodontic Surgery: Systematic Review and Meta-Analysis with Trial Sequential Analysis. Medicina.

[2] Setzer F.C., Shah S.B., Kohli M.R., Karabucak B., Kim S. (2010). Outcome of endodontic surgery: A meta-analysis of the literature—Part 1: Comparison of traditional root-end surgery and endodontic microsurgery. J. Endod..

[3] Kassenzahnärztliche Bundesvereinigung (KZBV) (2023). Statistische Basisdaten zur vertragszahnärztlichen Versorgung 2023. https://www.kzbv.de/kzbv2023-jahrbuch-web-ohnegoz.media.9083f41ba25e0a1dfbdf6b349f333c2b.pdf.

[4] von Arx T. (2011). Apical surgery: A review of current techniques and outcome. Saudi Dent. J..

[5] Kang M., Kim E. (2024). Euiseong Kim Healing Outcome after Maxillary Sinus Perforation in Endodontic Microsurgery. J. Korean Dent. Sci..

[6] Von Arx T., Bolt S., Bornstein M.M. (2021). Neurosensory Disturbances After Apical Surgery of Mandibular Premolars and Molars: A Retrospective Analysis and Case-Control Study. Eur. Endod. J..

[7] Chugal N., Assad H., Markovic D., Mallya S.M. (2024). Applying the American Association of Endodontists and American Academy of Oral and Maxillofacial Radiology guidelines for cone-beam computed tomography prescription: Impact on endodontic clinical decisions. J. Am. Dent. Assoc..

[8] Surya S., Barua A.N.D., Magar S.P., Magar S.S., Rela R., Chhabada A.K. (2022). Comparative Assessment of the Efficacy of Two-Dimensional Digital Intraoral Radiography to Three-Dimensional Cone Beam Computed Tomography in the Diagnosis of Periapical Pathologies. J. Pharm. Bioallied Sci..

[9] D’haese J., Ackhurst J., Wismeijer D., De Bruyn H., Tahmaseb A. (2017). Current state of the art of computer-guided implant surgery. Periodontology 2000.

[10] Tsokkou S., Konstantinidis I., Keramas A., Kiosis G., Skourtsidis K., Alexiou D., Keskesiadou G.-N., Karachrysafi S., Papamitsou T., Chatzistefanou I. (2025). Comparative Analysis of Fully Guided and Free-Hand Orthognathic Surgery: Advancements, Precision, and Clinical Outcomes. Dent. J..

[11] Spille J., Helmstetter E., Kübel P., Weitkamp J.-T., Wagner J., Wieker H., Naujokat H., Flörke C., Wiltfang J., Gülses A. (2022). Learning Curve and Comparison of Dynamic Implant Placement Accuracy Using a Navigation System in Young Professionals. Dent. J..

[12] Gargallo-Albiol J., Barootchi S., Salomó-Coll O., Wang H.-L. (2019). Advantages and disadvantages of implant navigation surgery. A systematic review. Ann. Anat..

[13] Setzer F.C., Shi K.J., Zhang Z., Yan H., Yoon H., Mupparapu M., Li J. (2020). Artificial Intelligence for the Computer-aided Detection of Periapical Lesions in Cone-beam Computed Tomographic Images. J. Endod..

[14] Ganz S.D. (2025). Fully-Guided Placement of Dental Implants Utilizing Nasopalatine Canal Fixation in a Novel Rotational Path Surgical Template Design: A Retrospective Case Series. J. Dent..

[15] Hung M., Yevseyevich D., Khazana M., Schwartz C., Lipsky M.S. (2025). Charting New Territory: AI Applications in Dental Caries Detection from Panoramic Imaging. Dent. J..

[16] Firincioglulari M., Boztuna M., Mirzaei O., Karanfiller T., Akkaya N., Orhan K. (2025). Segmentation of Pulp and Pulp Stones with Automatic Deep Learning in Panoramic Radiographs: An Artificial Intelligence Study. Dent. J..

[17] Satapathy S.K., Kunam A., Rashme R., Sudarsanam P.P., Gupta A., Kumar H.S.K. (2024). AI-Assisted Treatment Planning for Dental Implant Placement: Clinical vs AI-Generated Plans. J. Pharm. Bioallied Sci..

[18] Negrete D., Lopes S.L.P.d.C., Barretto M.D.d.A., Moura N.B.d., Nahás A.C.R., Costa A.L.F. (2025). Artificial Intelligence and Dentomaxillofacial Radiology Education: Innovations and Perspectives. Dent. J..

[19] Aldahmash S.A., Price J.B., Mostoufi B., Griffin I.L., Dianat O., Tordik P.A., Martinho F.C. (2022). Real-time 3-dimensional Dynamic Navigation System in Endodontic Microsurgery: A Cadaver Study. J. Endod..

[20] Dianat O., Nosrat A., Mostoufi B., Price J.B., Gupta S., Martinho F.C. (2021). Accuracy and efficiency of guided root-end resection using a dynamic navigation system: A human cadaver study. Int. Endod. J..

[21] Gambarini G., Galli M., Stefanelli L.V., Di Nardo D., Morese A., Seracchiani M., De Angelis F., Di Carlo S., Testarelli L. (2019). Endodontic Microsurgery Using Dynamic Navigation System: A Case Report. J. Endod..

[22] Spille J., Bube N., Wagner J., Spille D., Birkenfeld F., Kübel P., Wiltfang J., Gülses A. (2023). Navigational exploration of bony defect mimicking a solid lesion of the mandible compared to conventional surgery by young professionals. J. Stomatol. Oral. Maxillofac. Surg..

[23] The Slicer Community 3D Slicer (Version 5.6.1) [Computer Software].

[24] Fedorov A., Beichel R., Kalpathy-Cramer J., Finet J., Fillion-Robin J.-C., Pujol S., Bauer C., Jennings D., Fennessy F., Sonka M. (2012). 3D Slicer as an Image Computing Platform for the Quantitative Imaging Network. Magn. Reson. Imaging.

[25] The Jamovi Project The Jamovi (Version 2.6) [Computer Software].

[26] Mezger U., Jendrewski C., Bartels M. (2013). Navigation in surgery. Langenbecks Arch. Surg..

[27] Spille J., Jin F., Behrens E., Açil Y., Lichtenstein J., Naujokat H., Gülses A., Flörke C., Wiltfang J. (2021). Comparison of implant placement accuracy in two different preoperative digital workflows: Navigated vs. pilot-drill-guided surgery. Int. J. Implants Dent..

[28] Varg E., Antal M., Major L., Kiscsatári R., Braunitzer G., Piffkó J. (2020). Guidance means accuracy: A randomized clinical trial on freehand versus guided dental implantation. Clin. Oral Implant Res..

[29] Block M.S., Emery R.W., Cullum D.R., Sheikh A. (2017). Implant Placement Is More Accurate Using Dynamic Navigation. J. Oral Maxillofac. Surg..

[30] Somogyi-Ganss E., Holmes H.I., Jokstad A. (2015). Accuracy of a novel prototype dynamic computer-assisted surgery system. Clin. Oral Implants Res..

[31] Buniag A.G., Pratt A.M., Ray J.J. (2021). Targeted Endodontic Microsurgery: A Retrospective Outcomes Assessment of 24 Cases. J. Endod..

[32] Hawkins T.K., Wealleans J.A., Pratt A.M., Ray J.J. (2020). Targeted endodontic microsurgery and endodontic microsurgery: A surgical simulation comparison. Int. Endod. J..

[33] Ahn S.-Y., Kim N.-H., Kim S., Karabucak B., Kim E. (2018). Computer-aided Design/Computer-aided Manufacturing-guided Endodontic Surgery: Guided Osteotomy and Apex Localization in a Mandibular Molar with a Thick Buccal Bone Plate. J. Endod..

[34] Giacomino C.M., Ray J.J., Wealleans J.A. (2018). Targeted Endodontic Microsurgery: A Novel Approach to Anatomically Challenging Scenarios Using 3-dimensional-printed Guides and Trephine Burs-A Report of 3 Cases. J. Endod..

[35] Martinho F.C., Rollor C., Westbrook K., Aldahmash S.A., Fay G.G., Rivera E., Parsa A., Price J.B., Tordik P.A. (2023). A Cadaver-based Comparison of Sleeve-guided Implant-drill and Dynamic Navigation Osteotomy and Root-end Resections. J. Endod..

[36] Kunzendorf B., Naujokat H., Wiltfang J. (2021). Indications for 3-D diagnostics and navigation in dental implantology with the focus on radiation exposure: A systematic review. Int. J. Implant Dent..

[37] Antony D.P., Thomas T., Nivedhitha M. (2020). Two-dimensional Periapical, Panoramic Radiography Versus Three-dimensional Cone-beam Computed Tomography in the Detection of Periapical Lesion After Endodontic Treatment: A Systematic Review. Cureus.

[38] Christiansen R., Kirkevang L.-L., Hørsted-Bindslev P., Wenzel A. (2009). Randomized clinical trial of root-end resection followed by root-end filling with mineral trioxide aggregate or smoothing of the orthograde gutta-percha root filling—1-year follow-up. Int. Endod. J..

[39] Kwak G.H., Kwak E.-J., Song J.M., Park H.R., Jung Y.-H., Cho B.-H., Hui P., Hwang J.J. (2020). Automatic mandibular canal detection using a deep convolutional neural network. Sci. Rep..

[40] Shah H., Hernandez P., Budin F., Chittajallu D., Vimort J.-B., Walters R., Mol A., Khan A., Paniagua B. (2018). Automatic quantification framework to detect cracks in teeth. Proc. SPIE Int. Soc. Opt. Eng..

[41] Wu Y., Wang F., Fan S., Chow J.K.-F. (2019). Robotics in Dental Implantology. Oral Maxillofac. Surg. Clin. N. Am..

[42] Xi S., Hu J., Yue G., Wang S. (2024). Accuracy of an autonomous dental implant robotic system in placing tilted implants for edentulous arches. J. Prosthet. Dent..

[43] Liu C., Wang X., Liu Y., Ma D., Wu Z., Wang H., Bai S., Zhao Y. (2025). Comparing the accuracy and treatment time of a robotic and dynamic navigation system in osteotomy and root-end resection: An in vitro study. Int. Endod. J..

